# Genotoxicity of Three Micro/Nanocelluloses with Different Physicochemical Characteristics in MG-63 and V79 Cells

**DOI:** 10.3390/jox12020009

**Published:** 2022-04-21

**Authors:** Célia Ventura, Catarina Marques, João Cadete, Madalena Vilar, Jorge F. S. Pedrosa, Fátima Pinto, Susete Nogueira Fernandes, Rafaela Raupp da Rosa, Maria Helena Godinho, Paulo J. T. Ferreira, Henriqueta Louro, Maria João Silva

**Affiliations:** 1 Department of Human Genetics, National Institute of Health Doutor Ricardo Jorge (INSA), Av. Padre Cruz, 1649-016 Lisbon, Portugal; catarina.a.c.marques@gmail.com (C.M.); joao.cadete@insa.min-saude.pt (J.C.); madalenafvilar@gmail.com (M.V.); fatima.pinto@insa.min-saude.pt (F.P.); henriqueta.louro@insa.min-saude.pt (H.L.); m.joao.silva@insa.min-saude.pt (M.J.S.); 2 Center for Toxicogenomics and Human Health (ToxOmics), NOVA Medical School-FCM, UNL, Rua Câmara Pestana, 6 Ed. CEDOC II, 1150-082 Lisbon, Portugal; 3 CIEPQPF, Department of Chemical Engineering, University of Coimbra, Pólo II, Rua Silvo Lima, 3030-790 Coimbra, Portugal; jorge_fsp@live.com.pt (J.F.S.P.); paulo@eq.uc.pt (P.J.T.F.); 4 CENIMAT/I3N, Department of Materials Science, NOVA School of Science and Technology (FCT NOVA), NOVA University Lisbon, Campus da Caparica, 2829-516 Caparica, Portugal; sm.fernandes@fct.unl.pt (S.N.F.); rr.rosa@fct.unl.pt (R.R.d.R.); mhg@fct.unl.pt (M.H.G.)

**Keywords:** nanofibrillated cellulose, nanocrystalline cellulose, nanotoxicology, cytotoxicity, genotoxicity, micronucleus assay

## Abstract

(1) Background: Nanocellulose is an innovative engineered nanomaterial with an enormous potential for use in a wide array of industrial and biomedical applications and with fast growing economic value. The expanding production of nanocellulose is leading to an increased human exposure, raising concerns about their potential health effects. This study was aimed at assessing the potential toxic and genotoxic effects of different nanocelluloses in two mammalian cell lines; (2) Methods: Two micro/nanocelluloses, produced with a TEMPO oxidation pre-treatment (CNFs) and an enzymatic pre-treatment (CMFs), and cellulose nanocrystals (CNCs) were tested in osteoblastic-like human cells (MG-63) and Chinese hamster lung fibroblasts (V79) using the MTT and clonogenic assays to analyse cytotoxicity, and the micronucleus assay to test genotoxicity; (3) Results: cytotoxicity was observed by the clonogenic assay in V79 cells, particularly for CNCs, but not by the MTT assay; CNF induced micronuclei in both cell lines and nucleoplasmic bridges in MG-63 cells; CMF and CNC induced micronuclei and nucleoplasmic bridges in MG-63 cells, but not in V79 cells; (4) Conclusions: All nanocelluloses revealed cytotoxicity and genotoxicity, although at different concentrations, that may be related to their physicochemical differences and availability for cell uptake, and to differences in the DNA damage response of the cell model.

## 1. Introduction

Cellulose is the most abundant biopolymer in nature, being the main structural constituent of the plant cell wall. It is found mainly in wood, cotton, hemp, flax and other plant-based materials, but can also be produced by algae, fungi and various bacteria (within the genera *Acetobacter, Agrobacterium, Rhizobium and Sarcina*) [1,2]. Over time, this material has been used for a variety of purposes, and, more recently, there has been a clear growth of its application due to the increased demand for renewable, biodegradable and environmentally sustainable products. Moreover, in the last decade, as the synthesis and production of nanomaterials (NMs) has expanded, nano-sized celluloses have also been produced, emerging as sustainable and renewable materials with a high economic impact. Nanocellulose has found diverse interesting applications in industry, including in paper, coatings, food, nanocomposite formulations and reinforcement, and innovative biomedical applications [3].

Within the biomedical field, applications of nanocellulose are numerous and progress has been increasingly visible [3,4,5,6]. Since micro/nanocelluloses are considered biocompatible, several investigations have been made regarding their use in areas such as wound healing [7,8,9,10], surgical suturing [11] and regenerative medicine, for instance, as a matrix for bone regeneration, scaffolds for tissue-engineered meniscus, blood vessels, ligaments or tendons [12,13,14,15,16]. CNF and CNC have been also investigated for long-lasting drug delivery systems for bioactive substances such as anti-inflammatory drugs, antibiotics and growth factors [17,18,19] or 3D cell culture scaffolds [20,21,22].

Nanocelluloses can be divided into different categories, according to the source, methodology and final characteristics, two of them being cellulose nanofibrils (CNFs and CMFs, also known as micro/nanofibrillated celluloses) and cellulose nanocrystals (CNCs or nanocrystalline celluloses) [23]. Regarding CNFs, they are generally obtained by the fibrillation of cellulosic wood fibres through intensive mechanical treatment, e.g., with a high-pressure homogenizer, typically preceded by a chemical or enzymatic treatment to reduce energy consumption [1,24,25,26]. One of the most effective chemical treatments for CNF production is the oxidation mediated by the 2,2,6,6-tetramethylpiperidine-1-oxyl radical (TEMPO), which introduces carboxylate and aldehyde functional groups into the cellulose fibres, facilitating the deconstruction process [27,28,29]. CNF dimensions may vary with the cellulose fibre source and production method but they typically range between 3–100 nm in width and have a length in the order of micrometers, with a length-to-width ratio (aspect ratio) usually greater than 10 (ISO/TS 20477:2017) [30]. The application of an enzymatic pre-treatment, in turn, results mainly in cellulose microfibrils (CMF) [31], since the deconstruction process is not so efficient [25,32,33]. CNCs derive from the crystalline regions of cellulose and are isolated from the cellulose amorphous domains of the nanofibrils by acid hydrolysis [34,35]. CNCs typically have an elongated rod-like shape, with a width of 3 to 50 nm and a length of several hundred nanometres, exhibiting an aspect ratio of usually less than 50, but greater than 5 (ISO/TS 20477:2017), a high degree of crystallinity and lower flexibility than CNFs [36,37,38,39].

In a biomedical application context, there are compounds present in plant nanocellulose that must be removed to avoid biocompatibility problems, such as lignin or hemicellulose, and therefore nanocellulose should be subjected to chemical processes to eliminate these compounds. However, these processes may increase their toxicity as they give rise to residual chemicals [5]. For this reason, in order to ensure biocompatibility when these compounds are present, it is recommended to analyse possible cytotoxic and inflammatory effects or to purify plant nanocelluloses [40]. In addition, even though some materials are characterised and approved as biocompatible in their macro- and microforms, their nanoforms can reveal toxicity because of their different capacity to cross cell membranes, interact with biomolecules and trigger cell responses, among other effects. Indeed, compared to their larger analogues of identical chemical composition, NMs display some distinct characteristics that may explain different biological effects. Among these, it is possible to highlight the higher surface-area-to-volume ratio, occurrence of agglomeration, interaction with proteins and uptake by different cellular components [41].

To date, several toxicological studies of nanocellulose, either in vivo or in vitro, but mostly in vitro, have been performed [42,43,44]. It is generally considered that nanocellulose uptake into cells is low, with most studies showing no significant cytotoxicity and genotoxicity [39]. Nevertheless, some studies have indicated that CNFs have genotoxic effects, both in vivo [45,46] and in vitro [47,48,49], and that CNCs can trigger a moderate to severe inflammatory reaction in macrophages, depending on the CNC functionalization [50,51,52]. These outcomes are apparently milder than those of other nanofibres, such as multiwalled carbon nanotubes (MWCNT) [39]. However, nanocellulose functionalization can affect its hydrophobicity, surface charge and surface chemistry, modifying its agglomeration, bioavailability, cellular uptake, interaction with the cell membrane and the subcellular components, and, thus, its toxicological effects.

This study was aimed at assessing the potential toxic and genotoxic effects of different nanocelluloses obtained from *Eucalyptus* kraft pulp, one CNF and one CMF obtained through TEMPO-mediated or enzymatic pre-treatments, respectively, and one CNC, in two mammalian cell lines. The two different cell lines, the Chinese hamster lung fibroblasts (V79 cells) and the human osteoblast-like (MG-63) cell line, represent two potential sites of contact of nanocelluloses in the human body, considering exposure by inhalation, e.g., in an industrial setting, and their application in biomedicine, e.g., in bone regeneration. Cytotoxicity was analysed using complementary methods, one based on the metabolic capacity of viable cells (MTT assay) and another one measuring the colony-forming ability of individual cells (clonogenic assay). Moreover, we present the results of the most widely recommended assay to assess genotoxicity, the cytokinesis-block micronucleus (CBMN) assay. In this assay, micronuclei, corresponding to chromosome fragments or whole chromosomes that remain in the cytoplasm of cytokinesis-blocked (binucleated) cells after mitosis, are scored under a bright field or fluorescence microscopy [53]. Moreover, nucleoplasmic bridges and nuclear buds, corresponding to structural chromosomal alterations or to DNA amplification, respectively, can also be scored [53]. To our knowledge, this is the first genotoxicity study of these three types of nanocelluloses performed with the micronucleus assay in these two cell lines.

## 2. Materials and Methods

### 2.1. Nanocellulose Production and Characterisation

All nanocelluloses were produced from industrial bleached *Eucalyptus globulus* kraft pulp. CNFs were obtained by TEMPO-mediated oxidation followed by mechanical treatment in a high-pressure homogenizer, according to a procedure described elsewhere [54,55]. For that, the pulp, previously refined at 4000 revolutions PFI, was mixed with TEMPO (0.016 g/g of fibres) and NaBr (0.1 g/g of fibres) in demineralized water and a NaClO solution (9.7% active chlorine) was slowly added (5 mM/g of fibre). The reaction was carried out for 2 h and kept at pH 10 by adding NaOH 0.1M. The fibres were thoroughly washed with demineralized water until final conductivity of the suspension was low (20 µS/cm). Then, the pre-treated fibres were passed through the homogenizer (GEA Niro Soavi Model Panther NS3006L) twice, at 500 bar and 1000 bar, to reduce the size. A different pre-treatment was applied to obtain CMFs using a commercial enzyme (Serzym; Sertec 20) in the reaction, which occurred under more mild conditions at pH 5 and 50 °C, before the mechanical treatment in a high-pressure homogenizer, according to the method described by Tarrés et al. (2016) [56]. CNCs were obtained from the kraft pulp using sulphuric acid (diluted to 62 wt. %, from 95–97%, Sigma-Aldrich, MO, USA, p.a.) and an acid solution/solid ratio of 8:1 from adaptations of the acid hydrolysis method described elsewhere [57,58]. The reactional process occurred at 55 °C, under mechanical stirring, and the mixture was quenched with ultrapure water after 75 min of reaction. Subsequently, several centrifugation cycles were used until CNCs were released into the supernatant (pH from 1.4–2.9). Next, the suspended CNCs were dialyzed against ultrapure water until a constant pH was reached. Finally, CNCs in their acid form (pH = 3.3 in suspension) were dried by a freeze-dying process (−45 °C, at 0.3 mbar, VaCO 2, Zirbus). The obtained acid hydrolysis yield was 44% (defined as mass of dry CNC/mass of dry kraft pulp).

Fibrillated celluloses were characterised as to their fibrillation yield, amount of carboxylic groups, degree of substitution, degree of polymerization and size. The fibrillation yield was determined in duplicate by submitting a 40 mL CNF/CMF suspension at 0.2 wt. % to centrifugation at 9000 rpm for 30 min (8965× *g*) in a Hettich Universal 32. The yield was calculated as the percentage of material remaining in the supernatant (*w*/*w*), corresponding to the nanofibrillated fraction of the sample [59]. The carboxyl content (C_COOH_) was determined by a conductometric titration according to a methodology reported elsewhere [50]. Briefly, a certain amount of CNF/CMF suspension, equivalent to 0.1 g dry weight, was well stirred and the pH was set to 3.0 with HCl, and then titrated to pH 11.0 with a 0.01 M NaOH solution. The carboxylate content was determined in triplicate from the conductivity curve. The degree of polymerization (DP) was calculated applying the Mark–Houwink equation with the parameters reported by Henriksson et al. (2008) [59,60], and based on intrinsic viscosity measurements. The intrinsic viscosity was measured using a standard capillary viscometer in 0.5 M cupricethylenediamine solution at 25 °C (ISO standard 5351:2010). The structure of the fibrils was assessed by polarised light optical microscopy using an Olympus BH-2 KPA microscope (Olympus Optical Co., Ltd., Tokyo, Japan) equipped with a high-resolution CCD colour camera (Olympus ColorView. In addition, field emission-scanning electron microscopy (FE-SEM) was performed using a Carl Zeiss, Merlin microscope with a Gemini II column on 20 g/m^2^ films prepared by air-drying of a 0.2% (*w*/*v*) nanocellulose suspension. Moreover, transmission electron microscopy (TEM) was also used. For that, TEM grids with a formvar carbon support film were placed on a drop of 10 μL of the sample for 5 min. Thereafter, the sample grids were washed in 10 drops of water and stained in 2% uranyl acetate for 5 min. The excess moisture was drained along the periphery using filter paper. The grids were examined using a FEI TEM equipped with a Velleta camera. TEM images were analysed with Image J, a public domain software, and the average of 10 measurements was considered as the diameter of each nanofibril. The zeta-potential of diluted nanocellulose suspensions (0.1 wt. %) was determined by electrophoretic light scattering (ELS) using a Zetasizer Nano ZS equipment equipped with a 532 nm laser (Malvern Instruments, Malvern, UK). CNCs were analysed by TEM and ELS, as previously described for CNFs and CMFs, and by atomic force microscopy (AFM) to attest the composition and dimensions of the CNC. CHS elemental determination was obtained using the Thermo Finnigan-CE Instruments Flash EA 1112 CHNS series analyser from solid CNC samples (2 mg). The results are an average of two independent measurements and two significant digits were considered. AFM images were acquired (Asylum Research MFP-3D standalone system in tapping mode, Santa Barbara, CA, USA) with silicone AFM probes, a scanning frequency of 300 kHz and k = 26 N/m. To obtain these images and allow the analysis of single nanoparticles, 20 µL of 0.01 wt. % CNC aqueous suspensions were deposited into a freshly cleaved mica surface (Muscovite Mica, V-5 from Electron Microscopy Sciences, PA, USA). Immediately before deposition, the suspension was dispersed, over an ice bath, using a UP400 S ultrasonic probe (6 mm probe tip, 400 W, 24 kHz, Hielscher Ultrasonics GmbH, Teltow, Germany) that generates a 3.2 Kj g^−1^ suspension. The droplets were allowed to dry at 60 °C until they were a constant weight and samples were kept in a desiccator until further use. Moreover, 4 × 4 µm^2^ height images were used to measure the average length of individual nanoparticles, as described by Saraiva et al. (2020) [58]. The width of each particle used in the length determination was returned from the height profile along its length, as described by Honoratos-Rios et al. (2018) [61]. Gwyddion open-source software (version 2.52, obtained from http://gwyddion.net/ accessed on 7 February 2022) was used for all determinations and a total of 100 nanoparticles were analysed.

A stock solution of 1.5 mg/mL nanocellulose in phosphate buffer (PBS) was prepared prior to cell exposure, from which the tested concentrations were diluted in the culture medium.

### 2.2. Cell Culture

The Chinese hamster lung fibroblasts, V79 cell line (ATCC^®^ CCL-93™, Manassas, VA, USA) was grown in Dulbecco’s Modified Eagle Medium (DMEM) (Invitrogen, Thermo Fisher Scientific, USA) and the human osteoblastic MG-63 cell line (ATCC^®^ CRL-1427™) was maintained in an RPMI 1640 medium (Invitrogen). Both cell media were supplemented with 1% penicillin/streptomycin (1000 U/mL and 10 mg/mL, Invitrogen), 10% foetal bovine serum (Invitrogen) and 1% fungizone (0.25 mg/mL, Invitrogen), and maintained in culture flasks at 37 °C, 5% CO_2_.

### 2.3. MTT Assay

The MTT assay was performed according to Bettencourt et al. (2020) [62]. Briefly, cells were plated in 96-well plates and allowed to attach for 24 h at 37 °C and 5% CO_2_. MG-63 cells were then exposed to 1.5, 3, 6.25, 12.5, 25 and 50 µg/cm^2^ nanocellulose (5–165 µg/mL), and V79 cells to 2.25, 4.5, 9, 18, 36 and 72 µg/cm^2^ nanocellulose (7.2–240 µg/mL) for 24 h or to sodium dodecyl sulphate (SDS; 1 µg/mL, Sigma) for 1 h (positive control). After washing with PBS, cells were incubated for 3 h with fresh growth medium containing 10% of the tetrazolium dye MTT solution (5 mg/mL, Calbiochem, Darmstadt, Germany). The MTT-containing medium was discharged and dimethyl sulfoxide (DMSO; Sigma) was added for 20 min under shaking. The absorbance was recorded at 570 nm against a reference filter set at 690 nm using a Multiscan Ascent spectrophotometer (Labsystems, Helsinki, Finland). The relative cell survival of exposed cultures was expressed as the ratio between the absorbance of the exposed and unexposed (negative control) cultures, assuming that the absorbance of the latter represents 100% cell survival. Results were obtained from three independent experiments, each using six replicate cultures.

### 2.4. Clonogenic Assay

The clonogenic assay was performed with V79 cells as described by Louro et al. (2019) [63]. Briefly, a very low density of V79 cells (50 cells) was plated in each well of a 6-well plate and allowed to attach for approximately 7 h, at 37 °C and 5% CO_2_. The cells were then exposed to 1.5, 3, 6.25, 12.5, 25 and 50 µg/cm^2^ nanocellulose (7.2–240 µg/mL) during 17 h at 37 °C, 5% CO_2_. For each experiment, negative (non-treated cells) and positive (0.2 µg/mL mitomycin C, Sigma) controls were included. At the end of this time, cells were washed with PBS (Gibco) and were incubated with fresh medium for a further 4 days, at 37 °C and 5% CO_2_ to allow colony formation. The wells were then washed with PBS, fixed with absolute methanol (Sigma) and stained with 10% Giemsa (Merck, Darmstadt, Germany). The number of colonies formed was counted and the cloning efficiency (CE) determined using the following equation [63]: CE = 100 × (no. colonies in negative control/no. of plated cells). The surviving fraction (SF) for each CNF concentration was calculated as follows: SF = (no. colonies formed after exposure/no. of plated cells) × CE/100. The cytotoxicity was determined based on the results from three independent experiments.

### 2.5. Micronucleus Assay

The cytokinesis-blocked micronucleus (CBMN) assay was carried out as described in OECD 487 (2016) [64]. Cells were exposed to a concentration range of 1.5, 3, 6.25 and 12.5 µg/cm^2^ of each nanocellulose (4.8–40 µg/mL in MG-63 cells and 7.2–57.6 µg/mL in V79 cells) for one cell cycle, and then cytochalasin B (Sigma) was added to each well at a final concentration of 6 µg/mL in fresh medium, being cells incubated for a further 1.5 to 2 cell cycles. For each experiment, negative (non-treated cells) and positive (0.1 µg/mL mitomycin C, Sigma) controls were included. Briefly, at the end of the treatment, cells were washed with PBS and, following detachment with trypsin-EDTA, cells were submitted to a hypotonic shock with KCl 0.1 M at 37 °C, centrifuged, fixed in a solution of absolute methanol:acetic acid (Sigma), and the pellet spread onto microscope slides. Slides were dried, stained with 4% Giemsa (Merck, Darmstadt, Germany) and air-dried at room temperature. Slides were scored under a bright field microscope for the presence of micronuclei (MN), using the criteria described by Fenech et al. (2007) [53]. At least 2000 binucleated cells from two independent cultures were blind-scored per treatment condition. In addition, nuclear buds and nucleoplasmic bridges were also scored in those binucleated cells and their mean frequency determined. The proportion of mono- (MC), bi- (BC) or multinucleated-cells (MTC) was calculated by scoring 1000 cells per treatment and the cytokinesis-blocked proliferation index (CBPI) was calculated as follows (OECD, 2016) [64]: CBPI = (MC + 2BC + 3MTC)/Total cells.

### 2.6. Statistical Analysis

Statistical comparisons of results of the clonogenic and MTT assays between treated and control cells were performed through a one-way analysis of variance (ANOVA) followed by Tukey’s multiple comparison test, after testing for data normality. Results from the micronucleus assay were analysed by the 2-tailed Fisher’s exact test and the CBPI index by the 2-tailed Student’s t-test. All analyses were performed with the IBM SPSS Statistics for Windows, Version 24.0 (IBM Corp., Armonk, NY, USA).

## 3. Results

### 3.1. Nanocellulose Characterisation

**Figure 1:**
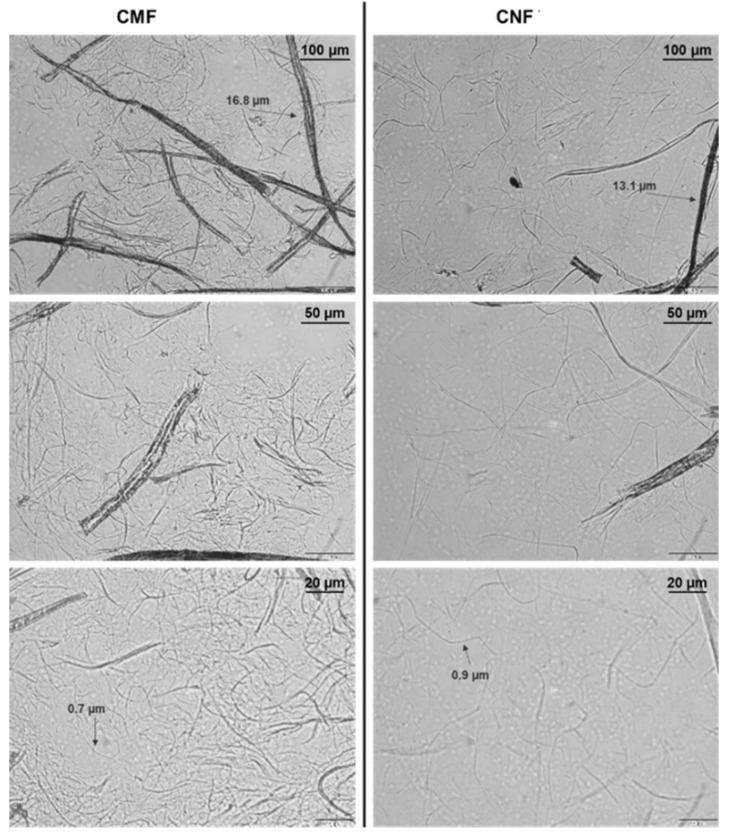
Polarised light microscopy images of the two cellulose micro/nanofibrils under study, obtained after enzymatic pre-treatment (CMF; **left**), and TEMPO-mediated oxidation (CNF; **right**) at different magnifications.

**Figure 2:**
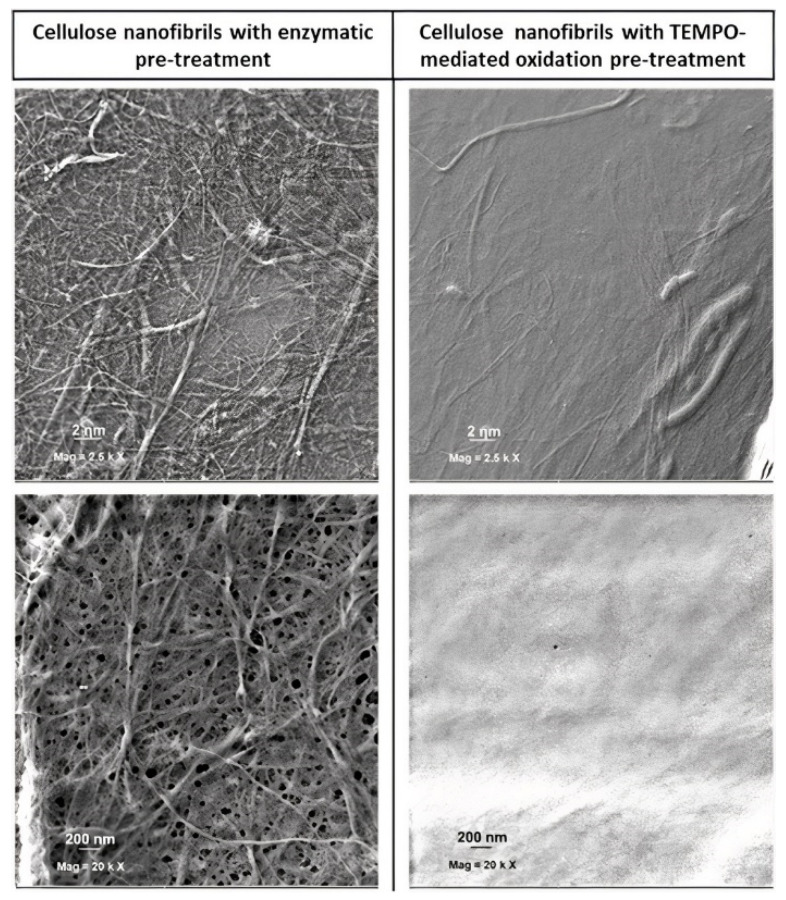
FE-SEM images of the two cellulose micro/nanofibrils obtained after enzymatic pre-treatment (CMF; **left**) and TEMPO-mediated oxidation (CNF; **right**) at different magnifications.

**Figure 3:**
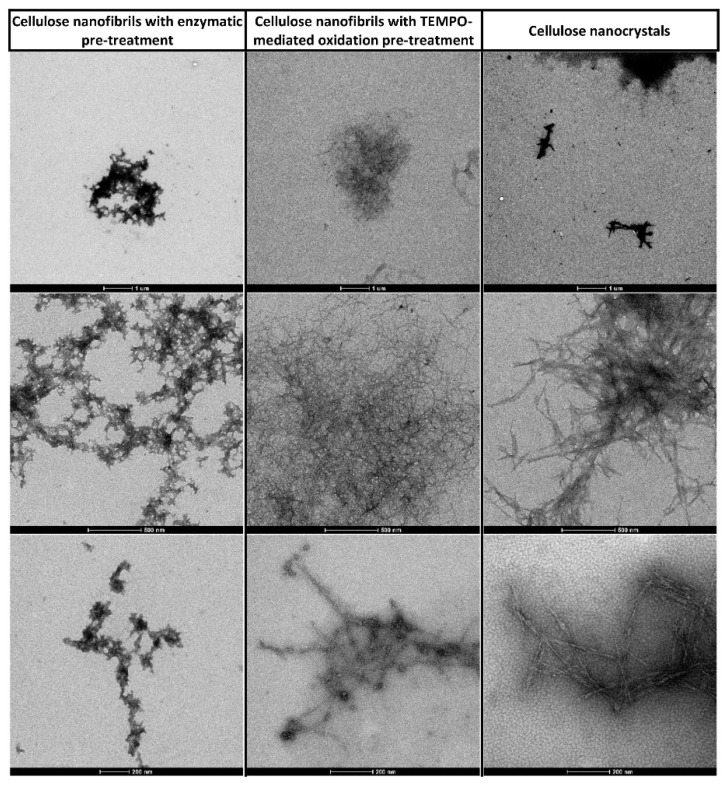
TEM images of the cellulose micro/nanofibrils obtained after enzymatic pre-treatment (CMF; **left**) and TEMPO-mediated oxidation (CNF; **centre**), and cellulose nanocrystals (CNC; **right**) at different magnifications (1 µm, 500 nm and 200 nm from top).

Bright-field microscopy images showed that CMF displayed non-fibrillated fibres together with differentiated fibrils with diameters near 1 µm and few smaller ones, which revealed diameters ranging 20 to 30 nm using FE-SEM. Concerning CNF, the number of fibrils with diameters of around 1 µm was much smaller, with most of the material being transformed into nanosized fibrils (Figure 1 and Figure 2). Nevertheless, FE-SEM of CNF and CMF did not allow the correct measure of the fibril’s diameter, particularly in the case of CNF due to the formation of an intricated nanofibre network (Figure 2), and TEM was used for all nanocelluloses under study (Figure 3).

The physicochemical characteristics of CNF and CMF, e.g., the number of carboxylic groups attached to the cellulose chain, the degree of polymerization, the intrinsic viscosity and fibrillation yield, the fibrils mean diameter (TEM) and the Z-potential after the TEMPO-mediated oxidation or enzymatic hydrolysis, are presented in Table 1, as well as the diameter and Z-potential of the CNCs that were also determined by the same methods. 

**Figure 4:**
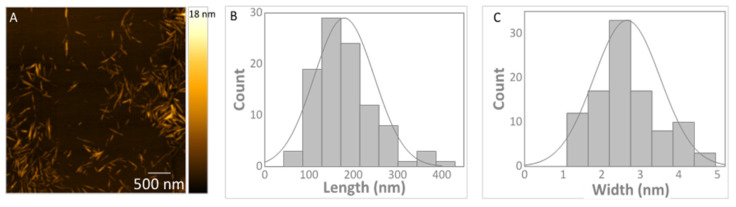
Example of an atomic force microscopy height image of CNC nanoparticles (**A**), and length (**B**) and width (**C**) distribution histograms obtained from AFM analysis.

Regarding the elemental analysis of CNCs, the results allowed inference of their chemical composition and the validation of the esterification reaction that occurs within the hydrolysis process. In this process, sulphate group esters are covalently linked to the surface of CNCs, allowing the stability of the nanoparticles in the suspension [60]. The wt. percentage of C, H and S achieved was 41.65, 6.06 and 0.63%, respectively. These values are in line with what was observed for samples produced from the same source and under similar hydrolysis conditions [58]. According to the formula, C_6_H_10_O_5_−(SO_3_), and considering equation S(%) = 100n × S/[6C + 10H + (5 + 3n)O + nS], 0.63% of sulphur is equivalent to 3.24 –OSO_3_H groups per 100 hydroglucose units. CNC nanoparticles present an average length of 180 ± 68nm and width of 3 ± 1 nm, which gives an aspect ratio of 60 ± 30. Some nanoparticles are presented in Figure 4A, alongside the length and width histograms (B and C, respectively) determined from AFM image analysis.

### 3.2. Cytotoxicity Assessment

**Figure 5:**
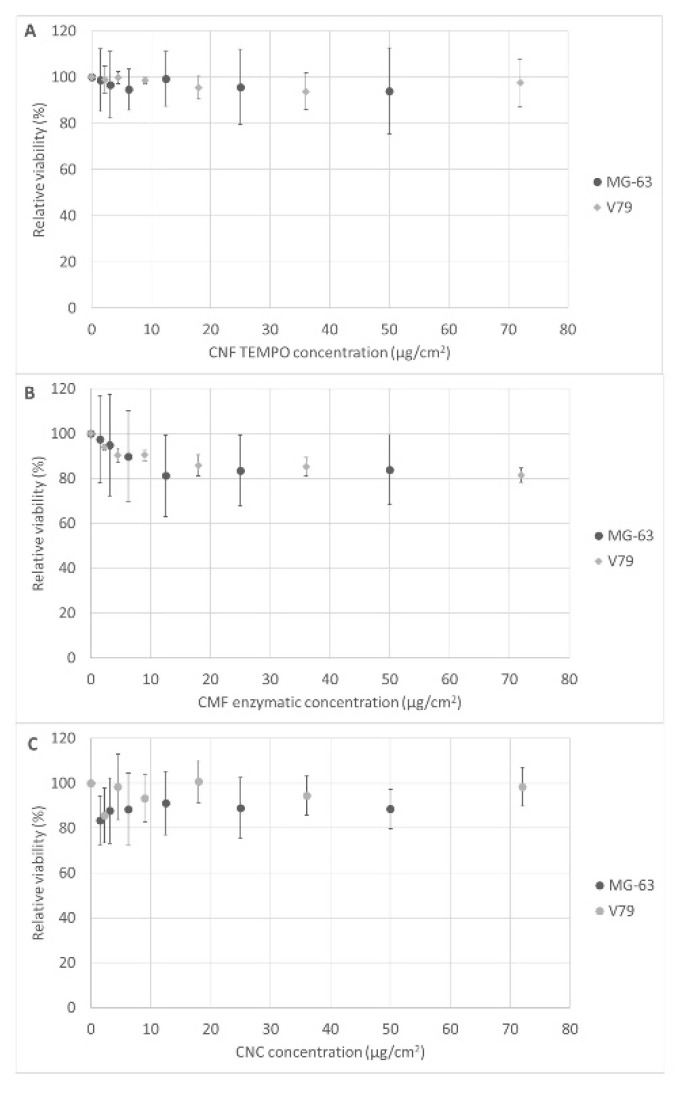
Cell viability relative to non-exposed control (MTT assay) after 24 h exposure to (**A**) TEMPO CNF, (**B**) enzymatic CMF, and (**C**) CNC, in MG-63 and V79 cells. Results are presented for each concentration tested as the mean ± SD of three independent experiments. Positive control (SDS 0.1%) yielded a relative viability that ranged from 1.7–8.1% in all experiments.

The results presented in Figure 5A–C refer to the MTT assay, and show that none of the nanocelluloses tested caused cytotoxicity in MG-63 or V79 cells after 24 h exposure.

**Figure 6:**
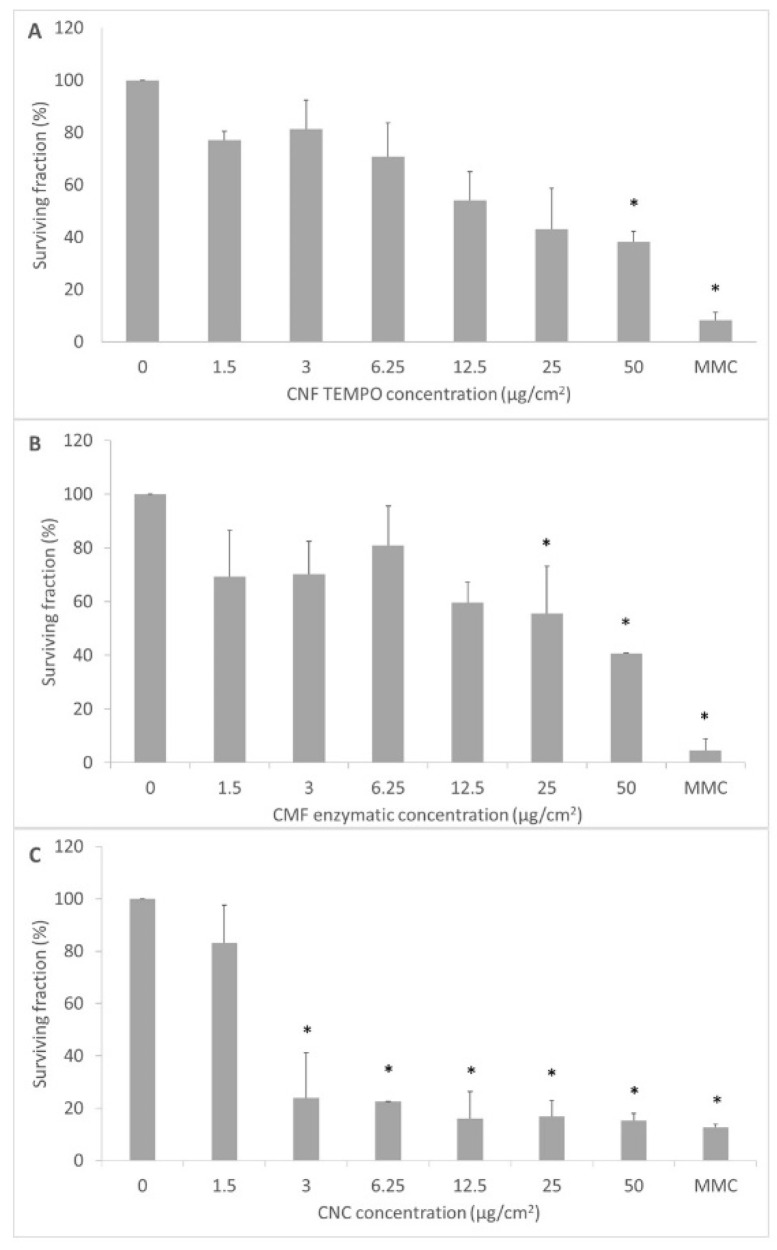
Results of the clonogenic assay after 17 h exposure to (**A**) TEMPO CNF, (**B**) enzymatic CMF, and (**C**) CNC in V79 cells, followed by 4 days resting. Results are presented as the mean ± SD of three independent experiments. MMC, mitomycin C. * *p* < 0.05.

The clonogenic assay was performed with V79 cells only, since MG-63 cells are unable to form colonies. The results presented in Figure 6A–C show that both CNF and CMF showed significant cytotoxicity at the highest doses tested (CNF, 50 µg/cm^2^, *p* = 0.05; CMF 25 µg/cm^2^; *p* = 0.04 and 50 µg/cm^2^; *p* = 0.003), and CNC had even a more pronounced effect, with clear cytotoxicity at all concentrations tested (*p* ≤ 0.001), except at the lowest one. Mitomycin C (0.2 µg/mL), used as a positive control, produced a highly significant cytotoxicity.

### 3.3. Genotoxicity Assessment

**Figure 7:**
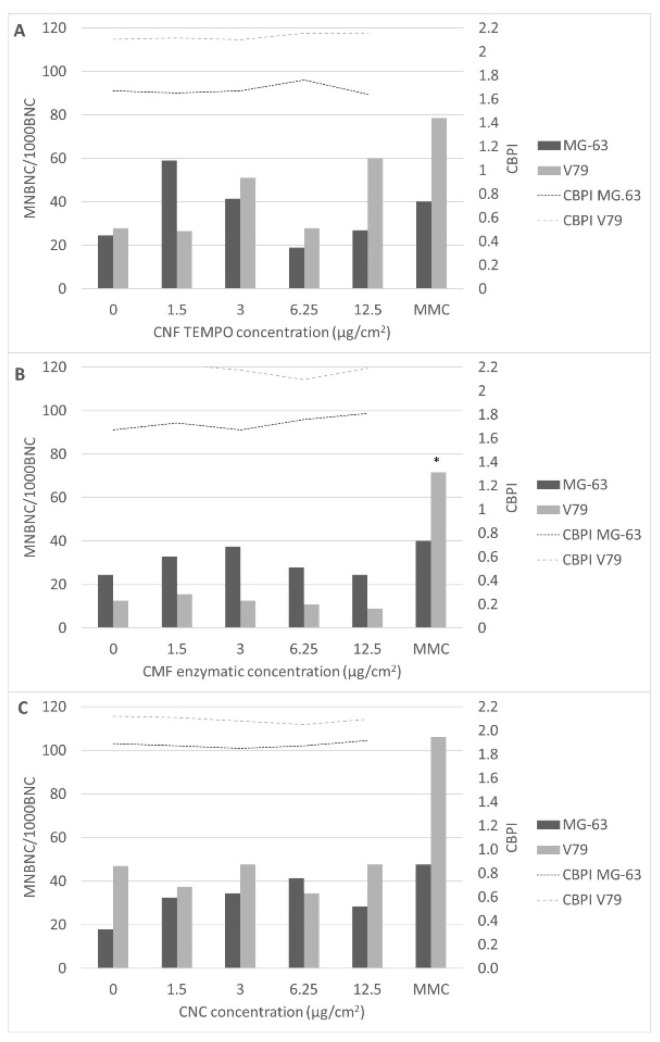
Frequency of micronucleated binucleated cells (MNBC) per 1000 binucleated cells (CBN) and CBPI values observed in MG-63 and V79 cells exposed to (**A**) TEMPO CNF, (**B**) enzymatic CMF, and (**C**) CNC. MMC, mitomycin C. * *p* < 0.05.

All nanocellulose samples were able to significantly raise the frequency of micronucleated cells in MG-63 cells, particularly at the lowest concentration range (1.5–3 µg/cm^2^; 4.8–9.6 µg/mL), whereas CNFs were the only nanocellulose that significantly induced micronucleus formation in V79 cells (Figure 7).

The cytokinesis block proliferation index (CBPI) that can be calculated in the CBMN assay is also presented in Figure 7. Cytostasis/cytotoxicity can be quantified through the CBPI that indicates the average number of cell cycles per cell during the period of exposure to cytochalasin-B, based on the number of mononucleated, binucleated and multinucleated cells from each treatment. It is noticeable that the CBPI values do not have a significant variation between any of the nanocellulose concentrations tested and the negative controls, showing that there was no significant delay in the cell cycle. 

**Figure 8:**
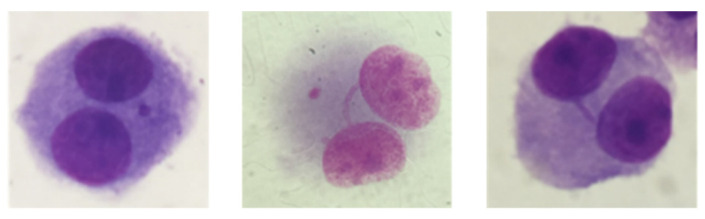
Representative images of MG-63 binucleated cells with micronuclei and/or nucleoplasmic bridges stained with Giemsa (Zeiss Axioscop 2 Plus microscope, Carl Zeiss AG, Jena, Germany, 400× and 1000×).

**Figure 9:**
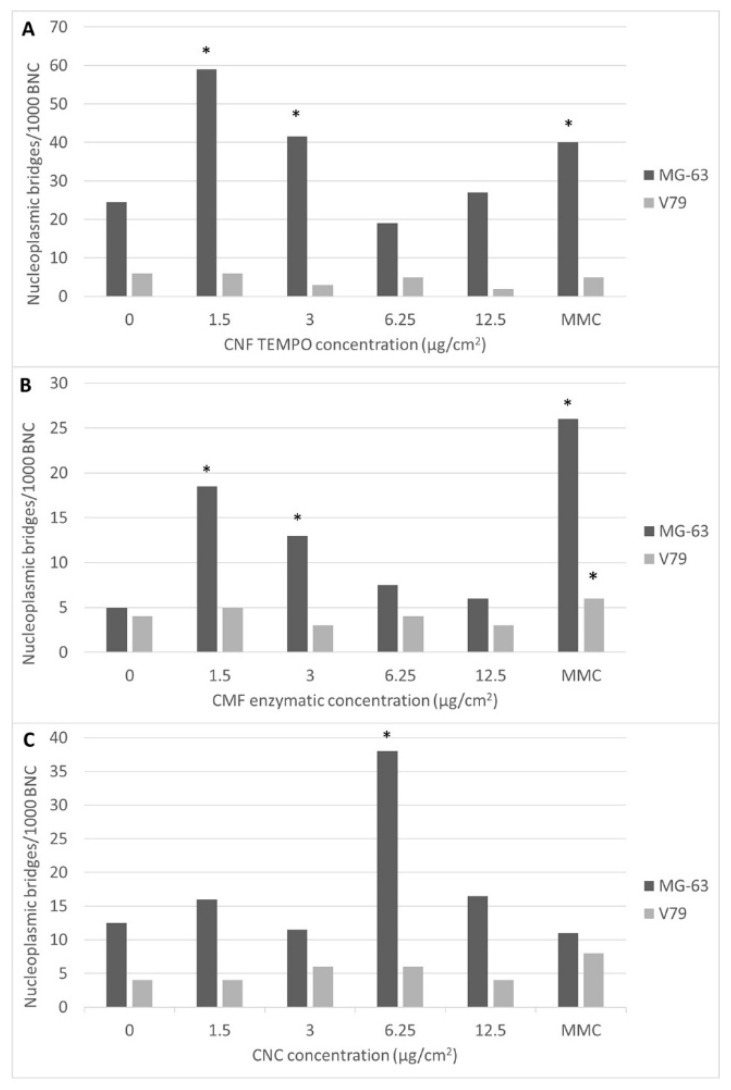
Frequency of binucleated cells (BNC) with nucleoplasmic bridges per 1000 BNC in MG-63 and V79 cells exposed to (**A**) TEMPO CNF, (**B**) enzymatic CMF, and (**C**) CNC. MMC, mitomycin C. * *p* < 0.05.

Besides micronuclei, both micro/nanofibres induced a significant number of nucleoplasmic bridges (Figure 8) in MG-63 cells, as compared to the controls (*p* ≤ 0.001), when exposed to the lowest concentrations tested (Figure 9A,B). CNCs also induced nucleoplasmic bridges in these cells when exposed to 6.25 µg/cm^2^ of CNCs (*p* ≤ 0.001) (Figure 9C). Moreover, nucleoplasmic bridges were not detected in V79 cells exposed to any type of nanocellulose. No significant induction of nuclear buds by the nanocelluloses studied was observed, irrespectively of the cell line used (data not shown).

## 4. Discussion

Toxicological assays should be performed for all newly developed nanomaterials to investigate their biosafety and biocompatibility before entering the market. In this study, two micro/nanocelluloses, one produced with TEMPO oxidation pre-treatment (CNFs) and other by enzymatic pre-treatment (CMFs), and cellulose nanocrystals (CNCs) were produced and characterised. All of them presented nanoscale diameters, but with different lengths (nm to µm) and different chemistries (carboxylic and sulphate groups), and all were shown to be negatively charged. Their characterisation revealed that they all comply with the requirements established in the international standards [30], and that they resemble the physicochemical characteristics of other nanocelluloses that has been reported in the literature by other groups, using the same production methods [39]. Firstly, a cytotoxicity assessment was used to screen potential cellular responses, and also to find out if the concentration range used to investigate the genotoxicity was adequate, i.e., if the concentrations tested were not too high to cause cytotoxic effects that could interfere with the genotoxicity results, as cytostasis or apoptosis. The results of the MMT assay indicate that neither CNFs, CMFs nor CNCs are cytotoxic to MG-63 or V79 cells, up to 50 µg/cm^2^ (165 µg/mL) and 72 µg/cm^2^ (240 µg/mL), respectively, after 24 h of exposure. This observation is supported by the CBPI calculated in the micronucleus assay, in that no significant CBPI decreases were estimated for the concentrations tested. The absence of a cytotoxic effect agrees with data from our study with TEMPO CNFs in A549 alveolar epithelial cells [45] and data from other toxicological studies of nanocellulose (reviewed in [39]). Nevertheless, after a longer period of time, i.e., 17 h of exposure followed by 4 days rest (clonogenic assay with V79 cells), all nanocelluloses revealed cytotoxicity, at least at the higher concentration tested (50 µg/cm^2^; 240 µg/mL). In particular, CNCs were cytotoxic at all but one concentration, i.e., the lowest one. One possible explanation may be that the sulphate groups that remain on the surface of CNCs as a result of cellulose hydrolysis with sulphuric acid (which results in cellulose esterification of hydroxyl groups with sulphate groups) may interfere with the cellular sulphate metabolism, a hypothesis that has to be investigated. Sulphate metabolism is essential for cell metabolism and signalling [65,66], and although CNC sulphate groups are only residual (0.63%), they may be blocking the sulphate transporters that are present in the cell membrane [66] over time, depriving cells from their normal influx. Another conceivable possibility is that the overdose of extracellular sulphate is being transported intracellularly over the time of exposure, cumulatively deregulating intracellular homeostasis. Nevertheless, both hypotheses will need further study to elucidate the possible role of sulphate groups in CNC cytotoxicity because, to our knowledge, this has never been addressed. 

These results also highlight the need for more toxicological studies using exposure times that are longer than those that are usually applied in in vitro toxicology. Moreover, these long-exposure studies better mimic chronic exposure scenarios, which are more common in real-life human exposure, for instance, in an occupational setting, and thereby deserve to be further explored. In fact, although the level of occupational exposure to nanocellulose appears to be irrelevant, as long as good working practices are implemented [67,68], and, currently, there are no occupational exposure limits (OEL) or recommended exposure limits (REL) for nanocellulose, its possible biopersistence in the lungs is of concern. There are still no studies on human exposure to nanocellulose, but a study carried out with artificial lung airway lining fluid and alveolar macrophage phagolysosomal fluid revealed a biopersistency of CNFs/CMFs and CNCs of up to 9 months, with the dominant mechanism of lung clearance possibly being mechanical clearance via the mucocilliary escalator to the gastrointestinal tract [69]. In addition, previous studies on cellulose indicated that, after one year of exposure, cellulose fibres are still present in rat lungs, showing that they have a higher biodurability in the lungs than ceramic fibres [70]. Therefore, the observation of cytotoxicity with exposure to CNFs/CMFs and CNCs for longer than 24 h demonstrates that more research is needed.

Regarding nanocellulose genotoxicity, the in vitro micronucleus assay is a reliable and predictive assay, in which an increased frequency of micronuclei, nucleoplasmic bridges and buds have been associated with an increased risk of cancer development, as physical injury to the DNA structure during cell division may increase genetic instability and may contribute to carcinogenicity [71,72,73]. Nevertheless, few nanocellulose genotoxicity studies in the literature have investigated chromosomal alterations. Catalán et al. 2015 exposed human bronchial epithelial BEAS-2B cells to a CNC (average length 135 ± 5 nm; width 7.3 ± 0.2 nm) in a concentration range of 2.5–100 µg/mL, and reported no induction of micronuclei [74]. A study with CNFs obtained from different sources reported that some were genotoxic in human lymphocytes and 3T3 cell mouse fibroblasts, but the presence of micronuclei was not investigated [75]. One in vivo study in female C57Bl/6 mice exposed to a single pharyngeal aspiration of 10, 40, 80 and 200 µg/mouse of CNFs (length 300–1000 nm; thickness 10–25 nm) for 24 h, showed DNA strand breaks in lung cells, but the micronuclei were only investigated in the bone marrow, where 24 h was insufficient to see an effect [45]. Increased DNA damage was also observed with an enzymatic non-carboxylated CNF in lung tissue of exposed mice, but, again, chromosomal damage was not investigated [46].

Since we previously observed that nanocelluloses displayed genotoxic properties, particularly at a low concentration range (1.5–3.12 µg/cm^2^; 4.8–9.6 µg/mL) [49], the present study focused the genotoxicity assessment at the lowest nanocellulose concentrations. Indeed, CNFs only induced micronuclei in MG-63 and V79 cells in the two lowest concentrations tested (1.5–3 µg/cm^2^). Similarly, the lowest concentration of CMFs was able to significantly raise the micronucleated MG-63 cells frequency (1.5 µg/cm^2^). Regarding the results in V79 cells, higher concentrations of CNF were necessary to elicit a significant increase in binucleated micronucleated cells, whereas no significant effects were induced by CMF. It is difficult to extrapolate these concentrations to the biomedical uses of nanocellulose by inhalation. In fact, many nanocelluloses that are being used for drug delivery systems are for oral intake and many biomedical applications are in the form of hydrogels, films and composites, where lung cells are not exposed to dispersed nanocellulose but to nanocellulose incorporated in structured systems [76,77].

Nevertheless, the results obtained following exposure of MG-63 cells to both micro/nano fibrillated cellulose make it imperative to carry out more studies using low dose-ranges, which are also more realistic regarding environmental or occupational chronic human exposure. One possible explanation for the absence of genotoxicity at the highest nanocellulose concentrations is that higher concentrations of NM are known to promote aggregation. If the NMs present in solution aggregate, the number of NMs that are dispersed and available to be taken up by the exposed cells is lower [78]. Thus, their effect on cells would be less with higher concentrations. This phenomenon has been already observed in previous studies with NMs [46,49]. Moreover, a possible explanation of the genotoxicity of CNCs in MG-63 cells at all concentrations may be that the possible cellular influx of the sulphate groups could cause oxidative damage to DNA [79,80,81,82], leading to double-strand breaks and structural chromosomal alterations, even without cellular uptake. As already mentioned, this hypothesis will have to be studied. CNCs presented no genotoxicity in V79 cells, similar to the negative results referred to above for CMFs, suggesting that this cell line is more resistant to the genotoxic effects of nanocelluloses than the MG-63 cell line.

Also relevant was the formation of nucleoplasmic bridges in MG-63 cells, particularly when exposed to CNFs and CMFs. Nucleoplasmic bridges are formed between the two nuclei of a binucleated cell and are typically associated with dicentric chromosomes [83,84]. However, nucleoplasmic bridges caused by cell internalization of nanofibres and their direct interference with the chromatin during mitosis has been proposed for MWCNTs [85]. There is evidence that CNTs may interfere with the organization of DNA, actin, microtubules and intermediate filaments, leading to cell division arrest and apoptosis [86], and may increase the frequency of disrupted centrosomes and abnormal mitotic spindles [87,88]. Therefore, CNFs and CMFs, being high aspect ratio nanofibres, can also have a similar effect. Cell uptake studies may contribute to identifying the mechanism by which they induce micronuclei and nucleoplasmic bridges in cells, and to evaluate if these two events can be related. Nevertheless, nucleoplasmic bridges were not observed in V79 cells.

The differences in the presence of micronuclei and nucleoplasmic bridges in MG-63 human osteoblasts and V79 mouse fibroblasts also demonstrate that different cell lines can provide dissimilar toxicological results. These can be due to different cell sensibilities. V79 cells, like many other rodent cell lines, are p53-deficient, and are known for giving misleading positive results with chemicals that do not induce genotoxicity or cancer in vivo, due to their higher susceptibility to cytotoxicity and genotoxicity, including a higher susceptibility to micronuclei induction [89]. By contrary, in this case, nanocelluloses are less genotoxic to V79 cells than to the p-53-competent human MG-63 cell line. One possible explanation could be that the impairment of p53 in V79 cells could lead to the death of V79 cells with high levels of DNA damage, and thereby, cells with chromosome alterations as micronuclei would no longer be present. Nevertheless, this hypothesis is not supported by the CBPI values, which measures cells proliferation kinetics, and by the mild to accentuated cytotoxicity observed only in the clonogenic assay with CNFs/CMFs and CNCs, respectively. It is also known that the culture medium can interact with the NMs being tested [90], forming a ‘corona’ that can affect their aggregation [91]. Given that both cell lines were grown with a comparable culture medium and supplemented with the same serum percentage, the latter is not a plausible explanation. Other factors related to the experimental design were similar in both cell lines, suggesting that the higher susceptibility of MG-63 cells to genotoxicity, as compared to V79 cells, is not due to differences in the analytical procedures. 

Therefore, for a more complete characterisation of nanocelluloses genotoxicity, genotoxicity assays targeting endpoints other than aneuploidy and clastogenicity are still needed [92]. Those endpoints may include either the induction of DNA lesions, which can be assessed, e.g., by the comet assay, or DNA mutations, which can be assessed by the hypoxanthine-guanine phosphoribosyltransferase (HPRT) test and thymidine kinase (TK) gene assay. Moreover, in vivo assays would also be useful, given that the use of cell lines does not fully mimic the complex interaction that occurs in the organism between various intracellular molecules, cell types and even organs [93]. Nevertheless, it should be stated that animal models should only be used when necessary for ethical reasons. Other relevant toxicological information on nanocelluloses can be obtained from ecotoxicological studies, such as the one carried out by Kovacs et al. (2010), where CNCs were found to have low potential toxicity and environmental risk to aquatic organisms, even under worst-case scenarios of CNC concentrations in the water, using extensive acute lethal toxicity testing and chronic sublethal tests with whole organisms [94].

Overall, the results presented here suggest that CNCs, the only rod-like nanofibers with sulphate group esters on their surface, are more cytotoxic and genotoxic than fibre-like CNFs/CMFs with carboxylic groups, and that CNFs are slightly more genotoxic than CMFs. Since all nanocelluloses are negatively charged, these differences can be related to their shape (aspect ratio) and surface chemistry. All of these features can influence nanocellulose aggregation, ‘corona’ formation and cellular uptake. These results are a contribution to the knowledge needed to enable a predictive toxicology approach, that is, to establish and use mechanisms and pathways of injury at a cellular and molecular level to prioritize the screening for adverse biological effects and health outcomes in vivo [95].

## 5. Conclusions

Since the use of nanocelluloses has been consistently increasing, it is of the utmost importance, both to the public and to occupational health, to ensure their safety for human health and the environment, in a cost-effective way. For this purpose, a first assessment of their potential toxicity before their large-scale production has been recommended to drive changes that can prevent toxicity. For that, it is necessary to combine the results from toxicity tests with knowledge of the physicochemical properties of nanocelluloses to better understand the most relevant properties behind their toxicity/genotoxicity. The results presented here support the suitability of the in vitro micronucleus assay to screen the genotoxicity of nanomaterials like nanocellulose. Our study indicates that all tested nanocelluloses are genotoxic in mammalian cells, CNFs (TEMPO pre-treatment) in both cell lines, and CMFs (enzymatic pre-treatment) and CNCs only in MG-63 cells. Nucleoplasmic bridges were associated with exposure to all types of nanocellulose, but only in MG-63 cells. Moreover, CNCs are more cytotoxic than the other two nanocelluloses at exposure times longer than 24 h. The molecular mechanisms of action of the three types of nanocelluloses must be further investigated in order to explain these findings, including the contribution of aggregation to micro/nanocellulose cell uptake and the possible effects of CNC sulphate groups in cell viability and DNA oxidative damage. The relevance of the cellular models in genotoxicity assessments is also highlighted. This knowledge will contribute to the assessment and prevention of human exposure to potentially hazardous nanocelluloses, and to a safe-by-design approach, enabling industries to develop innovative and safe materials without risk to human health. 

## Figures and Tables

**Table 1:** Chemical and physical characteristics of the cellulose micro/nanofibrils.

Sample	Fibrillation Yield (%)	C_COOH_ Content (µmol/g)	Intrinsic Viscosity (mL/g)	Degree of Polymerization	Mean Diameter (nm)	Zeta Potential (mV)
CNF TEMPO	100	1332	130	309	10.7 ± 1.9	−24.6 ± 1.0
CMF Enzymatic	4.9	143	618	1591	29.7 ± 7.3	−11.6 ± 1.0
CNC	NA	NA	NA	NA	19.7 ± 6.1	−17.3 ± 0.8

NA: not applicable.

## Data Availability

Data are contained within the article.
